# Supplementary material for: A novel strategy for community screening of SARS-CoV-2 (COVID-19): Sample pooling method
Source: PLoS One. 2020 Aug 28;15(8):e0238417. doi: 10.1371/journal.pone.0238417 (PMC7454965; doi:10.1371/journal.pone.0238417)
Supplement: S1 Table — (PDF) [file pone.0238417.s001.pdf]

S1 Table. Primers and probes for qRT-PCR, SARS-CoV-2 detection[a].

Gene target	Oligonucleotide	Sequence[b]
RNA-dependent RNA polymerase (RdRP)	RdRp_SARSr-F	5’-GTGARATGGTCATGTGTGGCGG-3’
	RdRp_SARSr-R	5’-CARATGTTAAASACACTATTAGCATA-3’
	RdRP_SARSr-P1	5’-FAM-CCAGGTGGWACRTCATCMGGTGATGC-BBQ-3’
	RdRp_SARSr-P2	5’-FAM-CAGGTGGAACCTCATCAGGAGATGC-BBQ-3’

[a] Oligonucleotide sequences were adapted from the WHO-Charité protocol [1].
[b] W is A/T; R is G/A; M is A/C; S is G/C. FAM: 6-carboxyfluorescein; BBQ: blackberry quencher.

References

1. Corman VM, Landt O, Kaiser M, Molenkamp R, Meijer A, Chu DKW, et al. Detection of 2019 novel coronavirus (2019-nCoV) by real-time RT-PCR. Eurosurveillance. 2020;25(3). Epub 2020/01/30. doi: 10.2807/1560-7917.ES.2020.25.3.2000045. PubMed PMID: 31992387; PubMed Central PMCID: PMCPMC6988269.
