# Supplementary material for: A novel strategy for community screening of SARS-CoV-2 (COVID-19): Sample pooling method
Source: PLoS One. 2020 Aug 28;15(8):e0238417. doi: 10.1371/journal.pone.0238417 (PMC7454965; doi:10.1371/journal.pone.0238417)
Supplement: S2 Table — (PDF) [file pone.0238417.s002.pdf]

S2 Table. Comparison of individual and pooled testing results in different nucleic acid extraction kit[a].

	Qiagen DNeasy Blood and Tissue Kit			Geneaid Viral Nucleic Acid Extraction Kit II		
**Sample ID**	**Individual[b]**	**Pooled[b]**	**C_T Difference**	**Individual[b]**	**Pooled[b]**	**C_T Difference**
H1 (IMU0094)	16.44 (0.36)	18.35 (0.41)	1.91	16.16 (0.25)	18.25 (0.19)	2.09
H2 (IMU0093)	17.84 (0.24)	18.61 (0.32)	0.76	18.56 (0.51)	19.25 (0.27)	0.69
M1 (IMU0047)	21.97 (0.80)	22.85 (1.02)	0.88	21.88 (0.76)	22.83 (0.45)	0.96
M2 (IMU0310)	27.13 (0.24)	27.73 (0.94)	0.59	25.45 (0.26)	28.41 (1.09)	2.96
L1 (IMU0254)	30.98 (0.60)	31.58 (0.44)	0.60	31.10 (0.89)	31.70 (1.02)	0.60
L2 (IMU0255)	34.61 (0.37)	35.40 (0.16)	0.79	35.75 (0.36)	34.95 (0.92)	0.80
Neg (IMU0006)	Not Detected	Not Detected	-	Not Detected	Not Detected	-
Neg (IMU0203)	Not Detected	Not Detected	-	Not Detected	Not Detected	-
Neg (IMU0357)	Not Detected	Not Detected	-	Not Detected	Not Detected	-
Neg (IMU0400)	Not Detected	Not Detected	-	Not Detected	Not Detected	-
Neg (IMU0498)	Not Detected	Not Detected	-	Not Detected	Not Detected	-
Neg (IMU0565)	Not Detected	Not Detected	-	Not Detected	Not Detected	-
Neg (IMU0651)	Not Detected	Not Detected	-	Not Detected	Not Detected	-
Neg (IMU0708)	Not Detected	Not Detected	-	Not Detected	Not Detected	-
Neg (IMU0931)	Not Detected	Not Detected	-	Not Detected	Not Detected	-
Neg (IMU0999)	Not Detected	Not Detected	-	Not Detected	Not Detected	-
Neg (IMU1109)	Not Detected	Not Detected	-	Not Detected	Not Detected	-
Neg (IMU1113)	Not Detected	Not Detected	-	Not Detected	Not Detected	-
Neg (IMU1042)	Not Detected	Not Detected	-	Not Detected	Not Detected	-
Neg (IMU1274)	Not Detected	Not Detected	-	Not Detected	Not Detected	-

[a] Nucleic acid extraction for each clinical specimen was performed using in both extraction kits according to the respective manufacturer's instructions. The qRT-PCR was performed in triplicates. The C_T values are expressed as mean (standard deviation).
[b] The volume of clinical specimen used for individual and pooled testing (10-sample pool) were 200 μL and 100 μL, respectively.
