# Supplementary material for: A novel strategy for community screening of SARS-CoV-2 (COVID-19): Sample pooling method
Source: PLoS One. 2020 Aug 28;15(8):e0238417. doi: 10.1371/journal.pone.0238417 (PMC7454965; doi:10.1371/journal.pone.0238417)
Supplement: S3 Table — (PDF) [file pone.0238417.s003.pdf]

S3 Table. Comparison of individual and pooled testing[b].

Detection of Severe Acute Respiratory Syndrome Coronavirus 2 (SARS-CoV-2) by qRT-PCR											
**Pool**	**Specimen ID**	**Pooled**	**Individual**	**Pool**	**Specimen ID**	**Pooled**	**Individual**	**Pool**	**Specimen ID**	**Pooled**	**Individual**
**1**	IMU1001	**–**	**–**	**4**	IMU1031	**–**	**–**	**7**	IMU1061	**–**	**–**
	IMU1002		**–**		IMU1032		**–**		IMU1062		**–**
	IMU1003		**–**		IMU1033		**–**		IMU1063		**–**
	IMU1004		**–**		IMU1034		**–**		IMU1064		**–**
	IMU1005		**–**		IMU1035		**–**		IMU1065		**–**
	IMU1006		**–**		IMU1036		**–**		IMU1066		**–**
	IMU1007		**–**		IMU1037		**–**		IMU1067		**–**
	IMU1008		**–**		IMU1038		**–**		IMU1068		**–**
	IMU1009		**–**		IMU1039		**–**		IMU1069		**–**
	IMU1010		**–**		IMU1040		**–**		IMU1070		**–**
**2**	IMU1011	**–**	**–**	**5**	IMU1041	**+**	**–**	**8**	IMU1071	**–**	**–**
	IMU1012		**–**		IMU1042		**–**		IMU1072		**–**
	IMU1013		**–**		IMU1043		**–**		IMU1073		**–**
	IMU1014		**–**		IMU1044		**–**		IMU1074		**–**
	IMU1015		**–**		IMU1045		**–**		IMU1075		**–**
	IMU1016		**–**		IMU1046		**–**		IMU1076		**–**
	IMU1017		**–**		IMU1047		**–**		IMU1077		**–**
	IMU1018		**–**		IMU1048		**–**		IMU1078		**–**
	IMU1019		**–**		**Pos. ctrl**		**+**		IMU1079		**–**
	IMU1020		**–**		IMU1050		**–**		IMU1080		**–**
**3**	IMU1021	**–**	**–**	**6**	IMU1051	**–**	**–**	**9**	IMU1081	**–**	**–**
	IMU1022		**–**		IMU1052		**–**		IMU1082		**–**
	IMU1023		**–**		IMU1053		**–**		IMU1083		**–**
	IMU1024		**–**		IMU1054		**–**		IMU1084		**–**
	IMU1025		**–**		IMU1055		**–**		IMU1085		**–**
	IMU1026		**–**		IMU1056		**–**		IMU1086		**–**
	IMU1027		**–**		IMU1057		**–**		IMU1087		**–**
	IMU1028		**–**		IMU1058		**–**		IMU1088		**–**
	IMU1029		**–**		IMU1059		**–**		IMU1089		**–**
	IMU1030		**–**		IMU1060		**–**		IMU1090		**–**

S3 Table (Cont'd). Comparison of individual and pooled testing[b].

Detection of Severe Acute Respiratory Syndrome Coronavirus 2 (SARS-CoV-2) by qRT-PCR											
**Pool**	**Specimen ID**	**Pooled**	**Individual**	**Pool**	**Specimen ID**	**Pooled**	**Individual**	**Pool**	**Specimen ID**	**Pooled**	**Individual**
**10**	IMU1091	–	–	**13**	IMU1121	–	–	**16**	IMU1151	–	–
	IMU1092		–		IMU1122		–		IMU1152		–
	IMU1093		–		IMU1123		–		IMU1153		–
	IMU1094		–		IMU1124		–		IMU1154		–
	IMU1095		–		IMU1125		–		IMU1155		–
	IMU1096		–		IMU1126		–		IMU1156		–
	IMU1097		–		IMU1127		–		IMU1157		–
	IMU1098		–		IMU1128		–		IMU1158		–
	IMU1099		–		IMU1129		–		IMU1159		–
	IMU1100		–		IMU1130		–		IMU1160		–
**11**	IMU1101	+	–	**14**	IMU1131	–	–	**17**	IMU1161	–	–
	**Pos. ctrl**		+		IMU1132		–		IMU1162		–
	IMU1103		–		IMU1133		–		IMU1163		–
	IMU1104		–		IMU1134		–		IMU1164		–
	IMU1105		–		IMU1135		–		IMU1165		–
	IMU1106		–		IMU1136		–		IMU1166		–
	IMU1107		–		IMU1137		–		IMU1167		–
	IMU1108		–		IMU1138		–		IMU1168		–
	IMU1109		–		IMU1139		–		IMU1169		–
	IMU1110		–		IMU1140		–		IMU1170		–
**12**	IMU1111	–	–	**15**	IMU1141	–	–	**18**	IMU1171	–	–
	IMU1112		–		IMU1142		–		IMU1172		–
	IMU1113		–		IMU1143		–		IMU1173		–
	IMU1114		–		IMU1144		–		IMU1174		–
	IMU1115		–		IMU1145		–		IMU1175		–
	IMU1116		–		IMU1146		–		IMU1049		–
	IMU1117		–		IMU1147		–		IMU1102		–
	IMU1118		–		IMU1148		–				
	IMU1119		–		IMU1149		–				
	IMU1120		–		IMU1150		–				

[a] The maximum testing capacity for the laboratory was 100 individual specimens per day; Group 1 – 9 were performed in parallel with 89 individual specimens on the first day, and Group 10 – 18 were performed in parallel with 86 individual specimens on the next day (N=175); Internal positive control (Pos. ctrl) was spiked into one of the pool for each day the screening was conducted.

[b] Interpretation of results of individual clinical specimen and pooled specimens on qRT-PCR assays; A pool or sample was considered positive for COVID-19 if the C_T value was less than or equal to 38. (+) Positive for COVID-19; (–) Negative for COVID-19.
