# Supplementary material for: A novel strategy for community screening of SARS-CoV-2 (COVID-19): Sample pooling method
Source: PLoS One. 2020 Aug 28;15(8):e0238417. doi: 10.1371/journal.pone.0238417 (PMC7454965; doi:10.1371/journal.pone.0238417)
Supplement: S1 Dataset — (PDF) [file pone.0238417.s004.pdf]

April 13th, 2020. IMU COVID-19 pooled testing results.

No	Group	Pooled samples (10 specimens), IMU Ref#	Real-Time RT-PCR result of pooled samples (C_T value)	Proceed with individual testing
1	A	1001 - 1010	Not Detected	No
2	B	1011 - 1020	Not Detected	No
3	C	1021 - 1030	Not Detected	No
4	D	1031 - 1040	Not Detected	No
5	**E**	1041 – 1050 (*Exclude sample 1049 due to insufficient specimen volume for pooled testing, replaced with **internal +ve control***)	29.89	Yes
6	F	1051 - 1060	Not Detected	No
7	G	1061- 1070	Not Detected	No
8	H	1071-1074	Not Detected	No
9	1049		Not Detected	-

Note: *Group H are composed of 4 samples pooled together, **1071 – 1074**.*
*Group **E** was spiked with one internal positive control (9 samples + 1 **internal +ve control**).*

Individual testing qRT-PCR results

Group E	IMU Ref#	C_T value	
		Pooled testing (1st qRT-PCR)	Individual (2nd qRT-PCR)
1	1041	29.89	Not Detected
2	1042		Not Detected
3	1043		Not Detected
4	1044		Not Detected
5	1045		Not Detected
6	1046		Not Detected
7	1047		Not Detected
8	1048		Not Detected
9	***internal +ve control***		29.16
10	1050		Not Detected

April 14th, 2020. IMU COVID-19 pooled testing results.

No	Group	Pooled samples (10 specimens), IMU Ref#	Real-time RT-PCR result of pooled samples (C_T value)	Proceed with individual testing
1	A	1075 – 1084	Not Detected	No
2	B	1085 – 1094 (*Exclude sample 1092 due to insufficient specimen volume for pooled testing, replaced with* ***sample 1175***)	Not Detected	No
3	**C**	1095 – 1104 (*Exclude sample 1102 due to insufficient specimen volume for pooled testing, replaced with* ***internal +ve control***)	30.18	Yes
4	D	1005 – 1114	Not Detected	No
5	E	1115 – 1124	Not Detected	No
6	F	1125 – 1134	Not Detected	No
7	G	1135 – 1144	Not Detected	No
8	H	1145 – 1154	Not Detected	No
9	I	1155 – 1164	Not Detected	No
10	J	1165 – 1174	Not Detected	No
11	1092		Not Detected	No
12	1102		Not Detected	No

Note: ***Group C*** *was spiked with one internal positive control (9 samples + 1* ***internal +ve control****).*

<u>Individual testing qRT-PCR results</u>

Group C	IMU Ref#	C_T value	
		Pooled testing (1st qRT-PCR)	Individual (2nd qRT-PCR)
1	1095	30.18	Not Detected
2	1096		Not Detected
3	1097		Not Detected
4	1098		Not Detected
5	1099		Not Detected
6	1100		Not Detected
7	1101		Not Detected
8	***internal +ve control***		29.07
9	1103		Not Detected
10	1104		Not Detected

April 15th, 2020. IMU COVID-19 pooled testing results.

No	Group	Pooled samples (10 specimens), IMU Ref#	Real-time RT-PCR result of pooled samples (C_T value)	Proceed with individual testing
1	A	1176 – 1185	Not Detected	No
2	B	1186 – 1195	Not Detected	No
3	C	1196 – 1205	Not Detected	No
4	D	1206 – 1215	Not Detected	No
5	E	1216 – 1225	Not Detected	No
6	F	1226 – 1235	Not Detected	No
7	G	1236 – 1245	Not Detected	No
8	H	1246 – 1255	Not Detected	No
9	I	1256 – 1265	Not Detected	No
10	**J**	1266 – 1274 (*Sample 1275 was extracted individually, and replaced with* ***internal +ve control***)	29.55	Yes
11	1275		Not Detected	-

Note: ***Group J*** *was spiked with one internal positive control (9 samples + 1* ***internal +ve control****).*

Individual testing qRT-PCR results

Group J	IMU Ref#	C_T value	
		Pooled testing (1st qRT-PCR)	Individual (2nd qRT-PCR)
1	1266	29.55	Not Detected
2	1267		Not Detected
3	1268		Not Detected
4	1269		Not Detected
5	1270		Not Detected
6	1271		Not Detected
7	1272		Not Detected
8	1273		Not Detected
9	1274		Not Detected
10	***internal +ve control***		30.54

April 16th, 2020. IMU COVID-19 pooled testing results.

No	Group	Pooled samples (10 specimens), IMU Ref#	Real-time RT-PCR result of pooled samples (C_T value)	Proceed with individual testing
1	A	1276 – 1285	Not Detected	No
2	B	1286 – 1295	Not Detected	No
3	C	1296 – 1305	25.39	Yes
4	D	1306 – 1315	Not Detected	No
5	E	1316 – 1325	Not Detected	No
6	F	1327 – 1336	Not Detected	No
7	G	1337 – 1346	Not Detected	No
8	H	1347 – 1356	Not Detected	No
9	I	1357 – 1366	Not Detected	No
10	J	1367, 1368, 1369, 1370, 1371, 1372	Not Detected	No
11	**K**	1373, 1374, 1375, 1376, 1377, 1326, ***internal +ve control***	28.86	Yes

Note: ***Group K*** *was spiked with one internal positive control (6 samples + 1* ***internal +ve control****).*
Sigmoidal curve with C_T value was observed for Group C.

Individual testing qRT-PCR results

Group C	IMU Ref#	C_T value Pooled testing (1st qRT-PCR)	C_T value Individual (2nd qRT-PCR)
1	1296	25.39	Not Detected
2	1297		Not Detected
3	1298		24.98
4	1299		Not Detected
5	1300		Not Detected
6	1301		Not Detected
7	1302		Not Detected
8	1303		Not Detected
9	1304		Not Detected
10	1305		Not Detected

Group K	IMU Ref#	C_T value Pooled testing (1st qRT-PCR)	C_T value Individual (2nd qRT-PCR)
1	1373	28.86	Not Detected
2	1374		Not Detected
3	1375		Not Detected
4	1376		Not Detected
5	1377		Not Detected
6	1326		Not Detected
7	***internal +ve control***		28.47

April 17th, 2020. IMU COVID-19 pooled testing results.

No	Group	Pooled samples (10 specimens), IMU Ref#	Real-time RT-PCR result of pooled samples (C_T value)	Proceed with individual testing
1	A	1276 – 1285	Not Detected	No
2	B	1286 – 1295	Not Detected	No
3	C	1296 – 1305	Not Detected	No
4	D	1306 – 1315	Not Detected	No
5	E	1316 – 1325	Not Detected	No
6	F	1327 – 1336	Not Detected	No
7	G	1337 – 1346	Not Detected	No
8	H	1347 – 1356	Not Detected	No
9	**I**	1458 – 1466 (9 samples **+** ***internal +ve control***)	23.71	Yes
10	J	1467 – 1471	40.16	Yes
11	K	1472 – 1477	Not Detected	No

Note: *__Group I__ was spiked with one internal positive control (9 samples + 1 __internal +ve control__). A C_T value of __40.16__ was obtained for __Group J,__ but no sigmoidal curve was observed. Nevertheless, we proceeded with individual testing to ensure the specimen was true negative.*

Individual testing qRT-PCR results

Group I	IMU Ref#	C_T value	
		Pooled testing (1st qRT-PCR)	Individual (2nd qRT-PCR)
1	1458	23.71	Not Detected
2	1459		Not Detected
3	1460		Not Detected
4	1461		Not Detected
5	1462		Not Detected
6	1463		Not Detected
7	1464		Not Detected
8	1465		Not Detected
9	1466		Not Detected
10	***internal +ve control***		23.23

Group J	IMU Ref#	C_T value	
		Pooled testing (1st qRT-PCR)	Individual (2nd qRT-PCR)
1	1467	40.16	Not Detected
2	1468		Not Detected
3	1469		Not Detected
4	1470		Not Detected
5	1471		Not Detected

April 18th, 2020. IMU COVID-19 pooled testing results.

No	Group	Pooled samples (10 samples), IMU Ref#	Real-Time RT-PCR result of pooled samples (C_T value)	Proceed with individual testing
1	A	1478 – 1487	30.61	Yes
2	B	1488 – 1497	20.50	Yes
3	C	1498 – 1507	22.97	Yes
4	D	1508 – 1517	24.85	Yes
5	E	1518 – 1527	26.60	Yes
6	F	1528 – 1537	21.11	Yes
7	G	1538 – 1547	24.85	Yes
8	H	1548 – 1557	Not Detected	No
9	I	1558 – 1567	Not Detected	No
10	J	1568 – 1577	Not Detected	No
11	K	1578 – 1587	35.72	Yes
12	**L**	1588 – 1596<br>(9 samples + ***internal +ve control***)	26.48	Yes
13	M	1597 – 1605	19.94	Yes

Note: ***Group L*** *was spiked with one internal positive control (9 samples + 1* ***internal +ve control****). Sigmoidal curve with C_T value was obtained for Group A, B ,C, D, E, F, G, K, L, M (total of 10 groups). Proceeded with individual testing and qRT-PCR assay was repeated for these specimens.*

Individual testing qRT-PCR results

Please refer to next page.

Group	No.	IMU Ref#	Pooled testing (1st qRT-PCR)	Individual (2nd qRT-PCR)	Group	No.	IMU Ref#	Pooled testing (1st qRT-PCR)	Individual (2nd qRT-PCR)
A	1	1478	30.61	Not Detected	F	51	1528	23.71	Not Detected
	2	1479		Not Detected		52	1529		**22.83**
	3	1480		Not Detected		53	1530		Not Detected
	4	1481		Not Detected		54	1531		Not Detected
	5	1482		Not Detected		55	1532		Not Detected
	6	1483		**29.42**		56	1533		Not Detected
	7	1484		Not Detected		57	1534		Not Detected
	8	1485		Not Detected		58	1535		**22.12**
	9	1486		Not Detected		59	1536		**33.12**
	10	1487		**30.03**		60	1537		**29.76**
B	11	1488	20.50	Not Detected	G	61	1538	24.85	**30.01**
	12	1489		Not Detected		62	1539		Not Detected
	13	1490		Not Detected		63	1540		Not Detected
	14	1491		Not Detected		64	1541		**23.32**
	15	1492		**24.25**		65	1542		Not Detected
	16	1493		Not Detected		66	1543		Not Detected
	17	1494		**16.74**		67	1544		**33.93**
	18	1495		Not Detected		68	1545		Not Detected
	19	1496		Not Detected		69	1546		Not Detected
	20	1497		Not Detected		70	1547		**30.42**
C	21	1498	22.97	Not Detected	K	71	1578	35.72	Not Detected
	22	1499		Not Detected		72	1579		Not Detected
	23	1500		**26.34**		73	1580		Not Detected
	24	1501		**20.02**		74	1581		Not Detected
	25	1502		Not Detected		75	1582		Not Detected
	26	1503		Not Detected		76	1583		Not Detected
	27	1504		Not Detected		77	1584		Not Detected
	28	1505		**30.08**		78	1585		Not Detected
	29	1506		Not Detected		79	1586		**31.61**
	30	1507		**27.03**		80	1587		Not Detected
D	31	1508	24.85	**23.79**	L	81	1588	26.48	Not Detected
	32	1509		Not Detected		82	1589		**32.91**
	33	1510		**29.13**		83	1590		Not Detected
	34	1511		Not Detected		84	1591		Not Detected
	35	1512		**23.47**		85	1592		Not Detected
	36	1513		Not Detected		86	1593		Not Detected
	37	1514		**30.54**		87	1594		Not Detected
	38	1515		**32.13**		88	1595		Not Detected
	39	1516		Not Detected		89	1596		Not Detected
	40	1517		Not Detected		90	***internal +ve ctrl***		23.04
E	41	1518	26.60	**33.56**	M	91	1597	19.94	**29.11**
	42	1519		Not Detected		92	1598		**19.03**
	43	1520		**23.81**		93	1599		**28.04**
	44	1521		Not Detected		94	1600		Not Detected
	45	1522		Not Detected		95	1601		Not Detected
	46	1523		**28.79**		96	1602		**30.40**
	47	1524		Not Detected		97	1603		**34.79**
	48	1525		**36.14**		98	1604		Not Detected
	49	1526		**23.49**		99	1605		**28.89**
	50	1527		Not Detected					

April 19th, 2020. IMU COVID-19 pooled testing results

Group	Pooled samples (5 specimens), IMU Ref#	Real-Time RT-PCR result of pooled samples (C_T value)	Proceed with individual testing
**1**	1606 – 1610	Not Detected	No
**2**	1611 – 1615	Not Detected	No
**3**	1616 – 1620	Not Detected	No
**4**	1621 – 1625	Not Detected	No
**5**	1626 – 1630	Not Detected	No
**6**	1631 – 1635	Not Detected	No
**7**	1636 – 1640	Not Detected	No
**8**	1641 – 1645	Not Detected	No
**9**	1646 – 1650	Not Detected	No
**10**	1651 – 1655	Not Detected	No
**11**	1656 – 1660	Not Detected	No
**12**	1661 – 1665	Not Detected	No
**13**	1666 – 1670	Not Detected	No
**14**	1671 – 1675	Not Detected	No
**15**	1676 – 1680	Not Detected	No
**16**	1681 – 1685	Not Detected	No
**17**	1686 – 1690	Not Detected	No
**18**	1691 – 1695	Not Detected	No
**19**	1696 – 1700	Not Detected	No
**20**	1701 – 1705	Not Detected	No
**21**	1706 – 1710	Not Detected	No
**22**	1711 – 1715	Not Detected	No
**23**	1716 – 1720	Not Detected	No
**24**	1726 – 1725	Not Detected	No
**25**	1726 – 1730	Not Detected	No
**26**	1731 – 1735	Not Detected	No
**27**	1736 – 1740	Not Detected	No
**28**	1741 – 1744<br>(4 samples + ***internal +ve control***)	22.08	Yes

Note: ***Group 28*** *was spiked with one internal positive control (4 specimens + 1* ***internal +ve control****).*

<u>Individual testing qRT-PCR results</u>

Group 28	IMU Ref#	C_T value	
		**Pooled testing (1st qRT-PCR)**	**Individual (2nd qRT-PCR)**
1	1741	22.08	Not Detected
2	1742		Not Detected
3	1743		Not Detected
4	1744		Not Detected
5	***internal +ve control***		22.32

April 21st, 2020. IMU COVID-19 pooled testing results

**No**	**Group**	**Pooled samples (10 samples), IMU Ref#**	**Real-Time RT-PCR result of pooled samples (C_T value)**	**Proceed with individual testing**
1	A	1745 – 1754	Not Detected	No
2	B	1755 – 1764	Not Detected	No
3	C	1765 – 1774	Not Detected	No
4	D	1775 – 1784	Not Detected	No
5	E	1785 – 1794	Not Detected	No
6	F	1795 – 1799 (5 samples)	Not Detected	No
7	G	1800 – 1809	Not Detected	No
8	H	1810 – 1819	Not Detected	No
9	I	1820 – 1829	Not Detected	No
10	J	1830 – 1839	Not Detected	No
11	K	1840 – 1849	Not Detected	No
12	**L**	1850 – 1854 (5 samples + ***internal +ve control***)	20.48	Yes
	External +ve control		28.34	-

*Note: **Group F** was a pooled sample with 5 samples (1795 – 1799). **Group L** was spiked with one internal positive control (5 samples + 1 **internal +ve control**).*

<u>**Individual testing qRT-PCR results**</u>

**Group L**	**IMU Ref#**	**C_T value**	
		**Pooled testing (1st qRT-PCR)**	**Individual (2nd qRT-PCR)**
1	1850	20.48	Not Detected
2	1851		Not Detected
3	1852		Not Detected
4	1853		Not Detected
5	1854		Not Detected
6	***internal +ve control***		20.34

April 22nd, 2020. IMU COVID-19 pooled testing results

Group	Pooled samples (10 samples), IMU Ref#	Real-Time RT-PCR result of pooled samples (C_T value)	Proceed with individual testing
1	1855 - 1863	Not Detected	No
2	1865 – 1874	Not Detected	No
3	1875 – 1884	Not Detected	No
4	1885 – 1894	Not Detected	No
5	1895 – 1904	Not Detected	No
6	1905 – 1914	Not Detected	No
7	1915 – 1924	Not Detected	No
8	1925 – 1934	Not Detected	No
9	1935 – 1944	Not Detected	No
10	1945 – 1954	Not Detected	No
11	1955 – 1964	Not Detected	No
12	1965 – 1974	Not Detected	No
13	1975 – 1984	Not Detected	No
14	1985 – 1994	Not Detected	No
15	1995 – 2004	Not Detected	No
16	2005 – 2014	Not Detected	No
17	2015 – 2024	Not Detected	No
18	2025 – 2034	Not Detected	No
19	2035 – 2044	Not Detected	No
20	2045 – 2054	Not Detected	No
21	2055 – 2064	Not Detected	No
22	2065 – 2074	Not Detected	No
**23**	2075 – 2083<br>(9 samples + ***internal +ve control***)	21.45	Yes

*Note: **Group 23** was spiked with one internal positive control (9 samples + 1 **internal +ve control**).*

Individual testing qRT-PCR results

Group 23	IMU Ref#	C_T value	
		Pooled testing (1st qRT-PCR)	Individual (2nd qRT-PCR)
1	2075	21.45	Not Detected
2	2076		Not Detected
3	2077		Not Detected
4	2078		Not Detected
5	2079		Not Detected
6	2080		Not Detected
7	2081		Not Detected
8	2082		Not Detected
9	2083		Not Detected
10	***internal +ve control***		21.07

April 23rd, 2020. IMU COVID-19 pooled testing results.

No	Group	Pooled samples (10 samples), IMU Ref#	Real-Time RT-PCR result of pooled samples (C_T value)	Proceed with individual testing
1	A	2084 – 2093	Not Detected	No
2	B	2094 – 2103	Not Detected	No
3	C	2104 – 2113	Not Detected	No
4	D	2114 – 2123	Not Detected	No
5	E	2124 – 2133	Not Detected	No
6	F	2134 – 2143	Not Detected	No
7	G	2144 – 2153	Not Detected	No
8	H	2154 – 2163	Not Detected	No
9	I	2164 – 2173	Not Detected	No
10	J	2174 – 2183	Not Detected	No
11	K	2184 – 2193	Not Detected	No
12	L	2194 – 2203	Not Detected	No
13	M	2204 – 2213	Not Detected	No
14	**N**	2214 – 2222 (9 samples + ***internal +ve control***)	20.92	Yes
15	O	2223 – 2232	Not Detected	No
16	**P**	2233 + ***external +ve control***	27.56	Yes

Note: ***Group N*** *was spiked with one internal positive control (9 samples + 1* ***internal +ve control****).* ***Group P*** *was spiked with one external positive control (1 sample + 1* ***external +ve control****)*

Individual testing qRT-PCR results

Group N	IMU Ref#	C_T value	
		Pooled testing (1st qRT-PCR)	Individual (2nd qRT-PCR)
1	2214	20.92	Not Detected
2	2215		Not Detected
3	2216		Not Detected
4	2217		Not Detected
5	2218		Not Detected
6	2219		Not Detected
7	2220		Not Detected
8	2221		Not Detected
9	2222		Not Detected
10	***internal +ve control***		20.99

Group P	IMU Ref#	C_T value	
		Pooled testing (1st qRT-PCR)	Pooled testing (1st qRT-PCR)
1	2233	27.56	Not Detected
2	***external +ve control***		27.32

April 24th, 2020. IMU COVID-19 pooled testing results.

No	Group	Pooled samples (10 samples), IMU Ref#	Real-Time RT-PCR result of pooled samples (C_T value)	Proceed with individual testing
1	A	2234 – 2243	Not Detected	No
2	B	2244 – 2253	Not Detected	No
3	C	2254 – 2263	Not Detected	No
4	D	2264 – 2273	Not Detected	No
5	E	2274 – 2283	Not Detected	No
6	F	2284 – 2293	Not Detected	No
7	G	2294 – 2303	Not Detected	No
8	H	2304 – 2313	Not Detected	No
9	I	2314 – 2323	Not Detected	No
10	J	2324 – 2333	Not Detected	No
11	K	2334 – 2343	Not Detected	No
12	L	2344 – 2353	Not Detected	No
13	M	2354 – 2363	Not Detected	No
14	N	2364 – 2373	Not Detected	No
15	O	2374 – 2383	Not Detected	No
16	**P**	2384 – 2392<br>(9 samples + ***internal +ve control***)	21.22	Yes
17	**Q**	2393 + ***external +ve control***	28.01	Yes

Note: ***Group P*** *was spiked with one internal positive control (9 samples + 1* ***internal +ve control****).* ***Group Q*** *was spiked with one external positive control (1 sample + 1* ***external +ve control****)*

Individual testing qRT-PCR results

Group P	IMU Ref#	C_T value	
		**Pooled testing (1st qRT-PCR)**	**Individual (2nd qRT-PCR)**
1	2384	21.22	Not Detected
2	2385		Not Detected
3	2386		Not Detected
4	2387		Not Detected
5	2388		Not Detected
6	2389		Not Detected
7	2390		Not Detected
8	2391		Not Detected
9	2392		Not Detected
10	***internal +ve control***		21.38

Group Q	IMU Ref#	C_T value	
		**Pooled testing (1st qRT-PCR)**	**Individual (2nd qRT-PCR)**
1	2393	28.01	Not Detected
2	***external +ve control***		27.32

April 25th, 2020. IMU COVID-19 pooled testing results.

No	Group	Pooled samples (10 samples), IMU Ref#	Real-Time RT-PCR result of pooled samples (C_T value)	Proceed with individual testing
1	A	2394 – 2403	Not Detected	No
2	B	2404 – 2413	Not Detected	No
3	C	2414 – 2423	Not Detected	No
4	D	2424 – 2433	Not Detected	No
5	E	2434 – 2443	Not Detected	No
6	F	2444 – 2453	Not Detected	No
7	G	2454 – 2463	Not Detected	No
8	H	2464 – 2473	Not Detected	No
9	I	2474 – 2483	Not Detected	No
10	J	2484 – 2493	Not Detected	No
11	K	2494 – 2503	Not Detected	No
12	L	2504 – 2513	Not Detected	No
13	M	2514 – 2523	Not Detected	No
14	N	2524 – 2533	Not Detected	No
15	O	2534 – 2543	Not Detected	No
16	P	2544 – 2553	Not Detected	No
17	Q	2554 – 2563	Not Detected	No
18	R	2564 – 2573	Not Detected	No
19	S	2574 – 2583	Not Detected	No
20	**T**	2584 – 2590 (7 samples + ***internal +ve control***)	21.36	Yes
21	***external +ve control***		27.51	-

Note: ***Group T*** *was spiked with one internal positive control (7 samples + 1* ***internal +ve control****).*

Individual testing qRT-PCR results

Group T	IMU Ref#	C_T value	
		**Pooled testing (1st qRT-PCR)**	**Individual (2nd qRT-PCR)**
1	2584	21.36	Not Detected
2	2585		Not Detected
3	2586		Not Detected
4	2587		Not Detected
5	2588		Not Detected
6	2589		Not Detected
7	2590		Not Detected
8	***internal +ve control***		21.24

April 27th, 2020. IMU COVID-19 pooled testing results.

No	Group	Pooled samples (10 samples), IMU Ref#	Real-Time RT-PCR result of pooled samples (C_T value)	Proceed with individual testing
1	A	2591 – 2600	Not Detected	No
2	B	2601 – 2610	Not Detected	No
3	C	2611 – 2620	36.1	Yes
4	D	2621 – 2630	Not Detected	No
5	E	2631 – 2640	Not Detected	No
6	F	2641 – 2650	Not Detected	No
7	G	2651 – 2660	Not Detected	No
8	H	2661 – 2670	Not Detected	No
9	I	2671 – 2680	Not Detected	No
10	J	2681 – 2690	Not Detected	No
11	K	2691 – 2700	Not Detected	No
12	L	2701 – 2710	Not Detected	No
13	M	2711 – 2720	Not Detected	No
14	N	2721 – 2730	Not Detected	No
15	O	2731 – 2740	Not Detected	No
16	**P**	2741 – 2745 (5 samples + ***internal +ve control***)	20.98	Yes
17	***external +ve control***		27.32	-

Note: ***Group P*** *was spiked with one internal positive control (5 samples + 1* ***internal +ve control****).*

Individual testing qRT-PCR results

Group C	IMU Ref#	C_T value	
		Pooled testing (1st qRT-PCR)	Individual (2nd qRT-PCR)
1	2611	36.1	Not Detected
2	2612		35.58
3	2613		Not Detected
4	2614		Not Detected
5	2615		Not Detected
6	2616		Not Detected
7	2617		Not Detected
8	2618		Not Detected
9	2619		Not Detected
10	2620		Not Detected

Group P	IMU Ref#	C_T value	
		Pooled testing (1st qRT-PCR)	Individual (2nd qRT-PCR)
1	2741	20.98	Not Detected
2	2742		Not Detected
3	2743		Not Detected
4	2744		Not Detected
5	2745		Not Detected
6	***internal +ve control***		20.12
