# Supplementary material for: A novel strategy for community screening of SARS-CoV-2 (COVID-19): Sample pooling method
Source: PLoS One. 2020 Aug 28;15(8):e0238417. doi: 10.1371/journal.pone.0238417 (PMC7454965; doi:10.1371/journal.pone.0238417)
Supplement: Attachment — Submitted filename: Response to Reviewers.docx [file pone.0238417.s005.docx]

***Journal Requirements:***

*When submitting your revision, we need you to address these additional requirements.*

*1. Please ensure that your manuscript meets PLOS ONE's style requirements, including those for file naming. The PLOS ONE style templates can be found at*

[*https://journals.plos.org/plosone/s/file?id=wjVg/PLOSOne_formatting_sample_main_body.pdf*](https://journals.plos.org/plosone/s/file?id=wjVg/PLOSOne_formatting_sample_main_body.pdf) *and*

[*https://journals.plos.org/plosone/s/file?id=ba62/PLOSOne_formatting_sample_title_authors_affiliations.pdf*](https://journals.plos.org/plosone/s/file?id=ba62/PLOSOne_formatting_sample_title_authors_affiliations.pdf)

The manuscript has been formatted accordingly.

*2. In your methods section, please disclose the sequences of the RT-PCR primers and ensure that their original source is cited, if applicable. Also, please briefly describe the recommendations of the WHO-Charité protocol in relation to the interpretation of results.*

The qRT-PCR primers and the original source have been included in S1 Table. A pool or sample was considered positive for COVID-19 if the C_T_ value was less than or equal to 38. We used the recommendations of the *WHO-Charité* protocol as a guideline for the interpretation of results for pooled testing.

*3. In your ethics statement and methods section, please clarify whether the biological samples used in your study were de-identified and fully anonymized before the researchers accessed them. If not, please clarify whether patients provided consent to use their samples or whether the ethics committee waived the need for consent.*

This study has obtained ethical clearance from the International Medical University Joint Research and Ethical Committee (EC/IRB Ref. No. 4.41/JCM-196/2020). All clinical specimens used in this study were fully anonymised and deidentified by assigning new laboratory reference number before the researchers can access them. The researchers are blinded from the patients’ information.

*4. Please include captions for your Supporting Information files at the end of your manuscript, and update any in-text citations to match accordingly. Please see our Supporting Information guidelines for more information:* [*http://journals.plos.org/plosone/s/supporting-information*](about:blank)*.*

The captions for all Supporting Information files have been added at the end of the manuscript.

**Reviewer 1**

*Personally, at a more detailed level, I am not convinced the argument made by the authors that pooling negative samples may generate false positives by increasing the viral load to a detectable limit (line 199 – 204 and line 271 – 280). I am more inclined to think the opposite may be more true, pooled samples tend to dilute the viral load. I think different explanation will need to be made.*

The explanation (line 218) has been removed and a study (Hogan, Sahoo & Pinsky, 2020) reporting similar findings (line 296-298) has been included in the manuscript.

*Additionally, in line 184, the authors referred to S1 Table, but the information is also available in table 1. Suggest including both Table 1 and S1 Table here.*

We have added Table 1 and S2 Table (line 199) accordingly.

**Reviewer 2**

*Comments for the author*

*The purpose of this paper is to develop a specimen pooling procedure that can be used for SARS-CoV-2 detection in samples. The procedure is validated with known positive specimens and also applied in clinical practice. Overall, I think this is an important contribution and should be published subject to minor revisions.*

*My comments about the paper are as follows.

1) “Pooling” goes by a few other different names: pooled testing and group testing. It would be good to include these names as key words or include somewhere else in the paper so that search engines can find this paper.*

Thank you for your suggestion. We have amended accordingly. We choose to use ‘pooled testing’ in the whole manuscript.

*2) The application of pooling to test for SARS-CoV-2 has many papers already, where most are available on pre-print servers like MedRxiv and ArXiv. The only peer-reviewed papers that I am aware of that use actual specimens are*

*• Abdalhamid et al. (American Journal of Clinical Pathology, 2020), https://academic.oup.com/ajcp/article/153/6/715/5822023
• Hogan et al. (Journal of the American Medical Association, 2020), https://jamanetwork.com/journals/jama/fullarticle/2764364
• Lohse et al. (Lancet Infectious Diseases, 2020), https://www.thelancet.com/journals/laninf/article/PIIS1473-3099(20)30362-5/fulltext
• Yelin et al. (Clinical Infectious Diseases, 2020),* [*https://academic.oup.com/cid/advance-article/doi/10.1093/cid/ciaa531/5828059*](https://academic.oup.com/cid/advance-article/doi/10.1093/cid/ciaa531/5828059)

*The Yelin et al. (2020) paper is already mentioned and differences between it and the submitted paper are described. The paper is now published, so its reference should be updated. The Hogan et al. (2020) paper is mentioned as well, but differences between it and the submitted paper need to be described. For the other two papers, you should include them, while also describing how their work differs with the submitted paper.*

Agreed. We have described the differences accordingly. We have also cited all the recommended articles in the manuscript.

(Line 284-292)

“During the Movement Control Order (MCO), Malaysia faced a shortage of both viral RNA extraction and RT-PCR kits. In addition, with more than 8000 results still pending during the MCO period, pooled testing was necessarily required. Pooled testing for COVID-19 has been practiced in several laboratories from different regions [7-10], with most laboratories practicing pooled testing for RT-PCR. Here we describe the pooled testing of 10 specimens starting from viral RNA extraction and followed by RT-PCR without decreasing the sensitivity of detection, even though we did not perform any modelling on group size prior to testing. We observed a threshold cycle (C_T_ value) difference of less than 3 between individual and pooled testing (S2 Table).”

(Line 307-314)

“Our finding is important as it has demonstrated that the nucleic acid extraction kits are interchangeable while the sensitivity remained high for COVID-19 diagnostic testing. Till today, many countries are still facing a shortage of COVID-19 testing kits. It is safe to believe that not every laboratory has unlimited supplies of the same testing kits throughout the testing period. Thus, improvisation of protocols may be necessary to overcome such a shortcoming. In general, the use of suggested pooled testing may require validation for effective COVID-19 diagnostic tests as protocols vary from laboratory to laboratory and manufacturer to manufacturer.”

*3) There are a number of separate investigations performed in the paper, and it was difficult to keep track of them all through initial readings. Overall, I see three main investigations:*

*a) “Preliminary testing of sample pooling method”,*

*b) “Comparison of sampling pooling and individual specimen testing”, and*

*c) “Prospective specimens screening using sample pooling method”*

*In the last paragraph of the introduction, one sentence could be given for each to explain their purpose and what is being done. This will help foreshadow to the reader what to expect in the remainder of the paper. Better linkages between these sections may be helpful too.*

We agree to the comment and we have added the paragraph (line 83-88) below in the introduction.

“As we did not perform pooled testing modelling and we are using different testing kits interchangeably, we performed a series of experiments to determine the framework of pooled testing in our laboratory. In preliminary pooled testing on clinical samples, we determined the sample size for pooled testing, followed by comparison of pooled and individual testing on clinical specimens to determine that the sensitivity of pooled testing is comparable to individual testing. We then use the validated pooled testing on the prospective clinical specimens.”

*4) Page 9 discusses the different volumes used. While it provides results for M2 and L2, what about the results for H1-H2, M1, and L2?*

For the investigation of different volumes used, we aim to investigate the detection limit (by volume) of the pooled testing by using positive controls with a lower range of viral loads. Both M2 and L2 were positive controls with moderate and low viral loads, respectively. From our experience, detecting samples with low viral loads was more essential to eliminate the possibility of getting false negative results. A false negative COVID-19 result can cause serious consequences and damage not only to the patient itself but also to public health. We have also included the data for H1, H2, M1 and L1 in Table 1.

*5) I am concerned about how standard deviations are represented in Tables 1 and 2 by “mean ± standard deviation”. While I have seen this representation in some other publications before, it is actually misleading because a reader may think all values are within a range of mean – standard deviation and mean + standard deviation or a reader may think this is some type of confidence interval. Better approaches for including standard deviations include “mean (standard deviation)” with a table caption or column header stating something like “mean (standard deviation) of …” or a separate column in the table for the standard deviations.*

Strongly agreed and thank you for your suggestion. We have amended accordingly.

*6) Page 11 states “n = 89 + 86” but it is not clear until page 15 why these numbers are given separately like this. I recommend giving a brief explanation on page 11 that these numbers represent tests performed over two separate days.*

Thank you for noticing this. We have included a brief explanation in the manuscript (line 234-237).

*7) In the Discussion section, I really liked seeing comments about prevalence and how this can be used to determine an appropriate group size. However, I think more guidance should be given about selecting group size. The statistical literature has many papers on pooling where guidance could be obtained. For example, these papers include Hitt et al. (Statistics in Medicine, 2019) and Kim et al. (Biometrics, 2007). The Abdalhamid et al. (2020) is the only paper on SARS-CoV-2 detection that I am familiar which addresses group size selection while also using actual samples.*

We mentioned in the manuscript that we did not perform pooled testing modeling prior testing. As we did not perform pooled testing modelling and we are using different testing kits interchangeably, we performed a series of experiments to determine the framework of pooled testing in our laboratory. Our in-house data showed that 5-sample pooling versus 10-sample pooling did not show any significant difference in C_T_ value, and thus we chose 10-sample pooling based on the low prevalence rate of positive COVID-19 in our specimens. A threshold cycle (C_T_ value) difference of less than 5 was observed between 5-sample and 10-sample pooling as shown in Table 1.

*8) The data corresponding to Figure 1 should be made available, perhaps in a format like what is given in Table 3 (each pool is displayed with the positive and negative indicators). Rather than an actual table within the paper, I think a data file would be sufficient. One reason to include the “raw” data rather than only the summarized data as in Figure 1 is that it allows readers to see how often multiple positives occur in the same pool. A summary perhaps could provide this same level of detail with
Number of positives Number of pools
0 1709
1 ___
2 ___
…
10 ___
rather than a data file or table like Table 3. One aspect that would be missing though is the time element. Given only three days of positives, this may not be too much of a loss.*

The data file corresponding to Figure 1 is now available under Supporting Information, S1 Dataset.
